# Supplementary material for: Origin of Ising magnetism in Ca3Co2O6 unveiled by orbital imaging
Source: Nat Commun. 2019 Nov 29;10:5447. doi: 10.1038/s41467-019-13273-4 (PMC6884600; doi:10.1038/s41467-019-13273-4)
Supplement: Supplementary file 1 — Supplementary Information [file 41467_2019_13273_MOESM1_ESM.pdf]

Supplementary Information for *"Origin of Ising magnetism in $Ca_3Co_2O_6$ unveiled by orbital imaging"*

Brett Leedahl,[1] Martin Sundermann,[1,2] Andrea Amorese,[1,2] Andrea Severing,[1,2] Hlynur Gretarsson,[1,3] Lunyong Zhang,[1] Alexander C. Komarek,[1] Antoine Maignan,[4] Maurits W. Haverkort,[5] and Liu Hao Tjeng[1]

[1]*Max Planck Institute for Chemical Physics of Solids, Nöthnitzer Straße 40, 01187 Dresden, Germany*

[2]*Institute of Physics II, University of Cologne, Zülpicher Straße 77, D-50937 Cologne, Germany*

[3]*PETRA III, Deutsches Elektronen-Synchrotron (DESY), Notkestraße 85, 22607 Hamburg, Germany*

[4]*Laboratoire CRISMAT, UMR 6508 CNRS-ENSICAEN, 6 bd Maréchal Juin, 14050 Caen Cedex, France*

[5]*Institute for Theoretical Physics, Heidelberg University, Philosophenweg 19, 69120 Heidelberg, Germany*

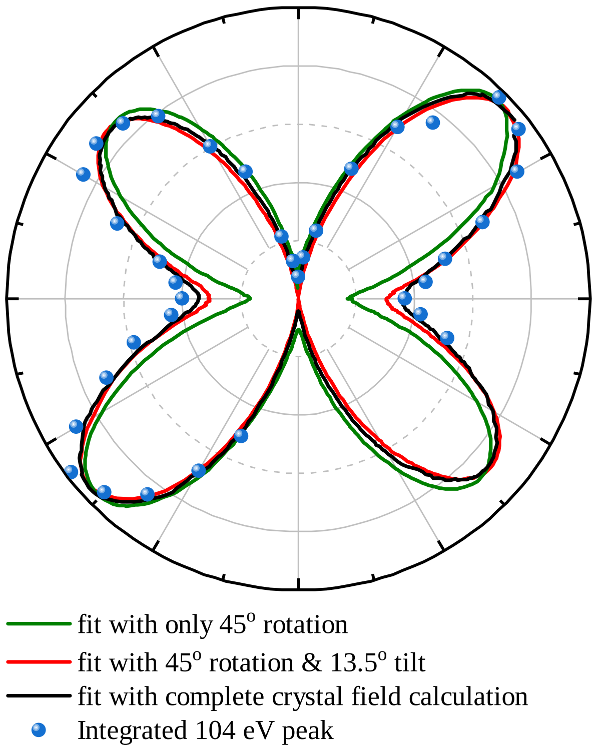

Supplementary Figure 1 . The experimental data points (blue) have been plotted along with three fits to show the effect of the crystal structure. The green curve is the fit including only that the two low-spin Co_{oct}^{3+} sites are alternatingly rotated 45° about the c-axis from one another. With only this consideration, the overall shape and orientation of the experimental data set are reasonably reproduced. Next, the red curve is the fit which also includes the 13.5° tilt off perpendicular from one another (see manuscript Fig. 1). The agreement with the experiment is now very good. The black curve (as displayed in Fig. 5a) is the fit which also includes the effect of the crystal fields associated with the slight deviation from perfect octahedral coordination of the Co_{oct} site. This effect is small, but nevertheless slightly improves the already very good agreement with experiment.

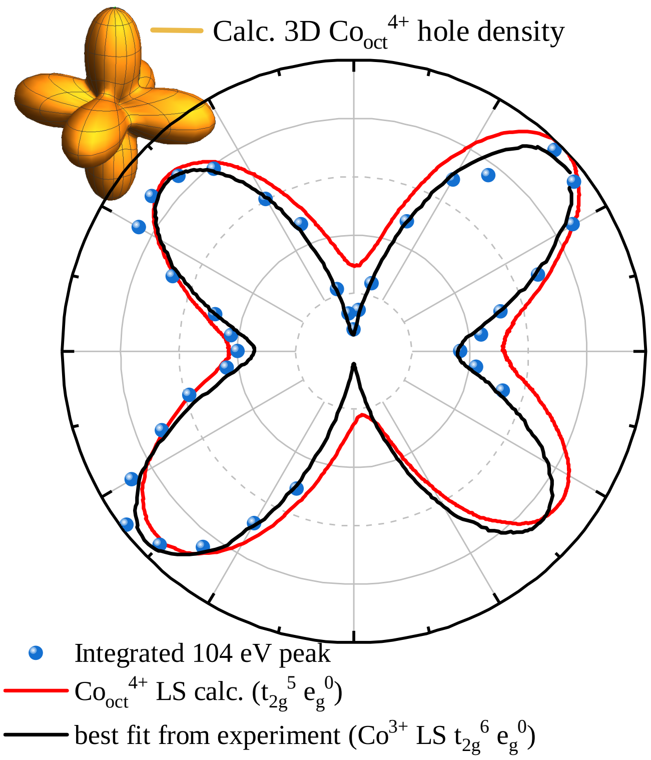

Supplementary Figure 2 . To convincingly show that our best solutions in Fig. 5 of the main manuscript are indeed a better description of the data than any alternative fit, we have calculated a commonly cited alternative solution for comparison [Ref. 7]. Plotted in red is the expected curve for the Co_{oct} site if it were Co^{4+} and low spin ($t_{2g}^{5}e_{g}^{0}$), which produces a lower quality fit to the experimental data points.

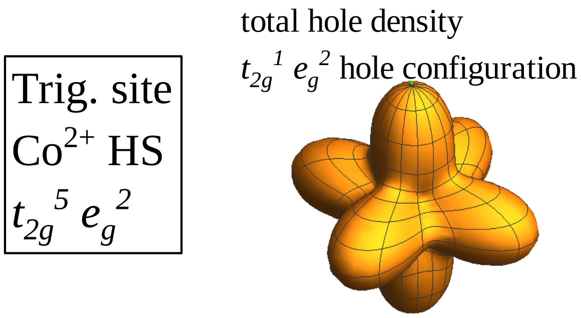

Supplementary Figure 3 . The Co_{trig} configuration that corresponds to the alternative choice for the Co_{oct} site shown in Fig. . No possible planar cut through this shape can reproduce the oval-like shape of the experimental data points shown in Fig. 5b of the main manuscript.

$A_{k,m}$	
$A_{0,0}$	0.00000
$A_{2,-2}$ =	0.30878+i*(0.06982)
$A_{2,-1}$ =	-0.58775+i*(0.20583)
$A_{2,0}$ =	0.13437+i*(-0.00000)
$A_{2,1}$ =	0.58775+i*(0.20583)
$A_{2,2}$ =	0.30878+i*(-0.06982)
$A_{4,-4}$ =	2.33651+i*(-0.00709)
$A_{4,-3}$ =	0.30052+i*(0.35238)
$A_{4,-2}$ =	-0.00191+i*(0.31177)
$A_{4,-1}$ =	-0.06235+i*(0.16160)
$A_{4,0}$ =	3.91300+i*(0.00000)
$A_{4,1}$ =	0.06235+i*(0.16160)
$A_{4,2}$ =	-0.00191+i*(-0.31177)
$A_{4,3}$ =	-0.30052+i*(0.35238)
$A_{4,4}$ =	2.33651+i*(0.00709)

Supplementary Table 1 . The potential due to the effect of the crystal field in a point charge model was calculated using the code in Ref. [1], wherein the only input is the crystal structure, following the $A_{k,m}$ nomenclature of Ref. [28]. These coefficients were then used to calculate a density matrix using the quantum many body script language *Quanty*[Ref. 27]. The density matrix contains coefficients for calculating the 3D charge and hole densities using the *Mathematica* toolboxes provided on *quanty.org*.
